# Neurodegenerative Susceptibility During Maternal Nutritional Programing: Are Central and Peripheral Innate Immune Training Relevant?

**DOI:** 10.3389/fnins.2020.00013

**Published:** 2020-02-04

**Authors:** Marcela Cárdenas-Tueme, Larisa Montalvo-Martínez, Roger Maldonado-Ruiz, Alberto Camacho-Morales, Diana Reséndez-Pérez

**Affiliations:** ^1^Departamento de Biología Celular y Genética, Facultad de Ciencias Biológicas, Universidad Autónoma de Nuevo León, San Nicolás de los Garza, Mexico; ^2^Departamento de Bioquímica, Facultad de Medicina, Universidad Autónoma de Nuevo León, San Nicolás de los Garza, Mexico; ^3^Centro de Investigación y Desarrollo en Ciencias de la Salud, Unidad de Neurometabolismo, Universidad Autónoma de Nuevo León, San Nicolás de los Garza, Mexico

**Keywords:** maternal programing, neurodegeneration, inflammation, immune training, cytokines

## Abstract

Maternal overnutrition modulates body weight, development of metabolic failure and, potentially, neurodegenerative susceptibility in the offspring. Overnutrition sets a chronic pro-inflammatory profile that integrates peripheral and central immune activation nodes, damaging neuronal physiology and survival. Innate immune cells exposed to hypercaloric diets might experience trained immunity. Here, we address the role of maternal overnutrition as a trigger for central and peripheral immune training and its contribution to neurodegeneration and the molecular nodes implicated in the Nod-like receptor protein 3 (NLRP3) inflammasome pathway leading to immune training. We propose that maternal overnutrition leads to peripheral or central immune training that favor neurodegenerative susceptibility in the offspring.

## Introduction

According to the World Health Organization, nearly 39% of adults were overweight in 2016, and 13% were obese. Epidemiological data from human catastrophes such as the Dutch famine (1944), the siege of Leningrad (1942–1944), the great Chinese famine (1958–1961), and the Överkalix study (1890-present) propose that changes in nutrient intake such as fasting or overnutrition during pregnancy and lactation are associated with metabolic and behavioral disorders in the offspring (Vaiserman, 2017). Maternal nutritional programing can influence offspring body weight, adiposity, and development of metabolic syndrome in diverse ways (Friedman, 2018; Patro Golab et al., 2018). For instance, nutritional programing adversely impacts both maternal and offspring health, increasing susceptibility to show metabolic abnormalities later in life such as obesity, dyslipidemia, type 2 diabetes (T2D) mellitus and hypertension, as well as behavioral disorders related to schizophrenia, autism, and compulsive eating (Shapiro et al., 2017; Friedman, 2018; Patro Golab et al., 2018; Derks et al., 2019).

Recent experimental evidence shows that exposure to hypercaloric diets programs the immune nodes that modulate metabolism and neuronal survival during embryogenesis. For instance, the immune response is involved in the balance and maintenance of an adequate metabolic state; however, conditions of altered metabolic demand promote a pro-inflammatory state (Christ et al., 2018). On this context, overnutrition might trigger an epigenetic process called “trained immunity” in innate immune cells, such as microglia in the central nervous system (CNS) and macrophages or natural killer cells in the periphery, which results in enhanced immune responses following infections or immune stimulation. The epigenetic mechanisms that mediate trained immunity are better understood in macrophages and monocytes, and a few have been described in microglia. Wendeln et al. (2018) demonstrated that microglia, just like peripheral macrophages, can be trained to confer long-lasting memory. In microglia, “trained immunity” is dependent on chromatin remodeling and activation of inflammatory signaling pathways (NF-κB, JNK, and ERK1/2). Also, by knocking down Hdac1/2 or Tak1 in long-lived CX3CR1 + cells (most of them microglia) cytokine levels were reduced, suggesting that epigenetic reprograming of inflammatory responses is key to accomplish immune training (Wendeln et al., 2018).

In this review, we address the role of maternal nutritional programing on central and peripheral immune training and crosstalk during positive metabolic scenarios, as well as their potential relevance in neurodegenerative susceptibility in the offspring.

## Central and Peripheral Immune System Communication

**FIGURE 1:**
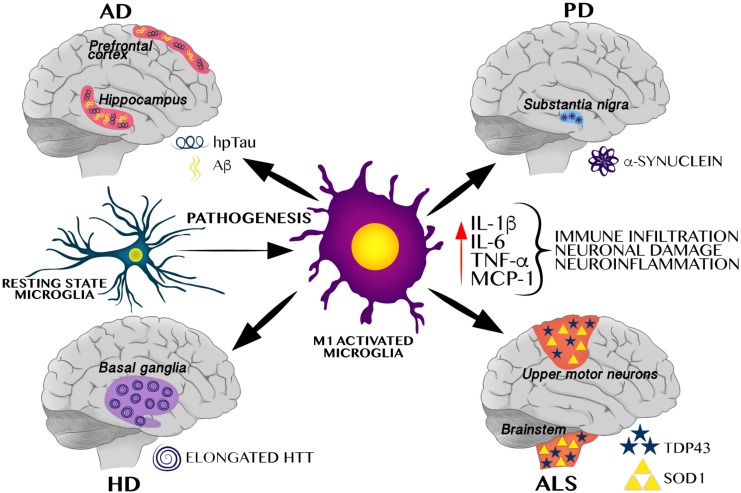
M1 Microglial activation induces a pro-inflammatory cytokines favoring neuroinflammation and protein aggregation-related neurodegenerative pathologies. M1 activated microglia induces a pro-inflammatory profile, including increase of IL-1β, TNF-α, MCP-1, and IL-6, all of these cytokines have been identified in different neurodegenerative pathologies including AD, PD, HD, and ALS. These neurodegenerative pathologies are characterized by selective protein aggregation, including amyloid beta (Aβ) and hyperphosphorylated Tau (hpTau) in AD, α-synuclein in PD, elongated huntingtin (HTT) in HD, and TAR DNA binding protein 43 (TDP43) and superoxide dismutase 1 (SOD1) in ALS.

The innate immune system is the first line of defense against pathogens and tissue damage; it includes physical barriers such as the skin, and specific cell types that modulate the inflammatory response such as macrophages, microglia, and complement proteins. The inflammatory response consists of an innate cellular system and humoral responses that occur during injury to restore homeostasis (Ciaccia, 2010). In this scenario, microglia, play the role of the innate immune system in the brain, infiltrating during early embryogenesis, coordinating neurogenesis, synaptic pruning, connectome establishment, and acting as major antigen-presenting cells (Colonna and Butovsky, 2017; Maldonado-Ruiz et al., 2017). In healthy brains, microglia remain in a ramified stated, and when activated, they enlarge their cell body, change to a phagocytic state, release pro-inflammatory cytokines, and increase antigen presentation and ROS production, leading to neuroinflammation (Colonna and Butovsky, 2017). As shown in Figure 1, under an altered physiological scenario, peripheral immune cells infiltrate the brain, become pro-inflammatory and secrete cytokines. This causes an exacerbated immune response by M1-activated microglia which leads to neuroinflammation, favoring protein aggregation and brain damage consistent with neurodegenerative pathologies (Colonna and Butovsky, 2017).

In the past years, the concept of the CNS as an immune-privileged site has changed, and interactions between the peripheral and central immune system have been demonstrated. Furthermore, these interactions are necessary to maintain homeostasis in the CNS, which was once thought to be impossible. For instance, the myeloid cells such as the macrophages and dendritic cells (DCs) in the meninges (dura, arachnoid, and pia mater), choroid plexus, and perivascular spaces in the CNS parenchyma can mount a robust protective and restorative response when necessary.

In addition, there is a strong relationship between inflammation and metabolic disorders in the immune response against infection. It is known that the immune response is involved in the balance and maintenance of an adequate metabolic state; however, it can have adverse effects under conditions of altered metabolic demand (Agrawal, 2014). In this way, positive energy balance during overnutrition promotes an inflammatory state in the CNS. For instance, saturated fatty acids from the diet are considered powerful candidates to trigger immune response in the peripheral system and in the CNS. However, how central immunity communicates with the peripheral immune response during overnutrition remains to be clarified (Maldonado-Ruiz et al., 2017). Based on this complex and delicate regulation, we next address how much the cells of the immune system can be influenced by selective diets.

## Maternal Programing by Exposure to High-Fat-High-Sugar Diets Activates Immunity and Regulates Neurodegenerative Susceptibility

Experimental data in animal models and humans have contributed to understand the effect of caloric overnutrition during pregnancy on immunity and neurodegenerative susceptibility later in life. The nervous system develops through a sophisticated and precise process from embryonic stages through puberty and adulthood. Proliferation, migration and differentiation of major brain cell types followed by rapid synaptogenesis integrate and establish a selective and efficient brain connectome. The immune system shares a time-dependent development and maturation with the nervous system during embryogenesis and post-natal periods (Estes and McAllister, 2016), which renders these systems extremely responsive to environmental factors, potentially modulating central and peripheral cross-talk and neurodegenerative susceptibility.

Initial experimental evidence in animal models and humans showing defects in glucose homeostasis such as T2D, have reported to contribute to neurodegeneration. For instance, rats fed a high-fat/glucose die develop insulin resistance and exhibit impaired spatial learning ability, reduced hippocampal dendritic spine density, and reduced long-term potentiation in the CA1 region due to glucotoxicity or altered insulin signaling (Stranahan et al., 2008). Moreover, Alzheimer’s disease (AD) transgenic mice models fed with a high-fat diet showed T2D-like peripheral insulin resistance but also more severe cognitive deficits compared to normoglycemic AD mice (Procaccini et al., 2016), demonstrating the possible interplay between defects in glucose metabolism and AD development. Of note, patients with T2D show two-fold higher risk to develop AD than normal subjects (Ott et al., 1999).

Experimental and clinical data support the hypothesis that inflammation itself might be a risk factor for neurodegeneration during AD (Ott et al., 1999; Bertram et al., 2007; Takahashi et al., 2007; Tartaglione et al., 2016). For instance, Ling Z. et al. (2004) and Ling Z. D. et al. (2004) exposed rats to LPS prenatally and then administered with a subtoxic dose of the dopaminergic (DA) neurotoxin rotenone (1.25 mg/kg per day for 14 days) when 16 months old. Of note, prenatal LPS and postnatal rotenone exposure exacerbate DA cell loss compared with the effects of single LPS or rotenone exposure. Also, initial genomic analysis of AD patients identified several risk genes that encode proteins involved in neural repair and remyelination and in the modulation of microglial responses, including phagocytosis (Bertram et al., 2007; Takahashi et al., 2007).

Obesity or maternal programing by exposure to high-fat-high-sugar diets in animal models and humans is in fact associated with overactivation of the immune system, triggering a process of chronic inflammation named “meta-inflammation” or “metabolic inflammation,” which ultimately links central and peripheral immune activation (O’Brien et al., 2017). For instance, maternal overnutrition of high-fat-high-sugar diets during pregnancy in murine models programs metabolic and hormonal settings that modulate neuronal development, axonal pruning, synaptic plasticity and connectome establishment during embryogenesis (Bilbo and Tsang, 2010; Morin et al., 2017; Mucellini et al., 2019). Also, mice born from obese dams have shown significant metabolic alterations including higher body fat, altered serum leptin levels, pancreatic islet hypertrophy, and lower expression of metabolism-related genes (Bilbo and Tsang, 2010; Gomes et al., 2018; Mucellini et al., 2019). In addition, human obesity is associated with the low-grade peripheral immune activation characteristic of an acute phase reaction, including C-reactive protein and serum amyloid A (SAA), an increase of cytokines, such as tumor necrosis factor (TNF-α), IL-1β, and IL-6 in serum, and an increase in white cell counts (Esser et al., 2014). In fact, systemic immune activation in psoriasis, a skin disorder, efficiently favors neuroinflammation (O’Brien et al., 2017). There are also other evidences in humans verifying that obesity is closely associated with neurodegenerative susceptibility including Alzheimer’s, Parkinson’s, and Huntington’s diseases and nervous system sclerosis (Procaccini et al., 2016), and an increase in the Body Mass Index in healthy mothers is negatively associated with white matter development in their offspring (Ou et al., 2015; Procaccini et al., 2016; Widen et al., 2018). These results suggest that positive energy balance such as during obesity or maternal overnutrition leads to neurodegenerative susceptibility, which potentially involves an immune activation pathway.

Initial reports of maternal programing by overnutrition using murine models identified sex-specific differences in fetal size and gene expression signatures in fetal-brains, showing that male offspring are the most affected. Importantly, the offspring displays a pro-inflammatory gene signature as the top regulated gene pathway (Edlow et al., 2016a). Later on, these results were confirmed in humans in a pilot study that analyzed the amniotic fluid from obese and lean pregnant women on the second trimester; in this study, 205 genes were found differentially regulated, where the lipid regulator, Apolipoprotein D, was the most upregulated gene (9-fold). Also, genes involved in apoptotic cell death were significantly downregulated particularly within pathways involving the cerebral cortex, such as the Serine/threonine kinase 24 ATPase (*STK24*). These biomarkers also correlated with the activation of the transcriptional regulators estrogen receptor, FOS, and STAT3, suggesting a pro-estrogenic, pro-inflammatory milieu (Edlow et al., 2014). In addition, this molecular signature was then confirmed in a prospective case-control study were 701 differentially regulated genes were identified, integrating a neurodegeneration gene signature, including Occludin (*OCLN*), Kinesin Family Member 14 (*KIF14*), and Nuclear receptor subfamily 2, group E, member 1 (*NR2E1*) (Edlow et al., 2016b). Upregulation of *OCLN* and *KIF14* genes have been previously associated to AD and vascular dementia in post-mortem human brain tissue (Romanitan et al., 2007), and in negative regulation of neuron apoptosis and regulation of myelination (Fujikura et al., 2013), respectively. Also, *NR2E1* is implicated in neurogenesis and neuronal differentiation modulating aggressive behavior and fear response (Young et al., 2002). Notably, all of these gene alterations also correlated with a pro-inflammatory signature of upregulated genes: chemokine (C-C motif) receptor 6 (CCR6), O-linked N-acetylglucosamine (GlcNAc) transferase (OGT), chemokine (C-C motif) receptor 2 (CCR2), caspase 4, apoptosis-related cysteine peptidase (CASP4), toll-like receptor 1 (TLR1), nucleoporin 107 kDa (NUP107), decapping enzyme, scavenger (D), among others (Edlow et al., 2016b). Together, maternal nutritional programing by overnutrition activates the central and peripheral immune systems that intimately communicate with each other to modulate neuroinflammation, thus increasing neurodegenerative susceptibility in offspring.

We will now discuss the experimental data addressing the potential role of maternal nutritional programing on the Nod-like receptor protein 3 (NLRP3) inflammasome pathway activation and its effects on neurodegeneration.

## Potential Role of the Nod-Like Receptor Protein 3 Inflammasome Pathway on Neurodegenerative Susceptibility by Nutrient Over Supply

The NLRP3- inflammasome pathway is linked to a danger-associated molecular pattern released from damaged or dying neurons that bind and activate the Toll-like receptor (TLR) –dependent myeloid differentiation primary response protein MyD88 (MYD88)–nuclear factor-κB (NF-κB) pathway. At first, the TLR-MYD88-NF-κB pathway positively produces pro-IL-1β and NLRP3 synthesis, activating a positive feedback loop. Then, negative stimuli, including changes in potassium efflux or reactive oxygen species, trigger the inflammasome assembly and processing of pro-IL-1β into IL-1β by caspase 1 activation (Heneka et al., 2018). Finally, the NF-κB transcription factor also regulates a variety of different processes, including stress response and a pro-inflammatory profile activation.

Initial reports by Christ et al. (2018) identified that murine models exposed to high-fat-high-sugar diets set an epigenetic program that primes B lymphocytes into an exacerbated pro-inflammatory phenotype. These, become much more responsive under physiologic stimuli which depend on the NLRP3-inflammasome pathway (Christ et al., 2018). Selective lipid species, such as palmitate and stearate, as well as, carbohydrates have been identified to activate the NLRP3-inflammasome pathway (Wen et al., 2011; Ann et al., 2018). For instance, saturated lipids from diet intake such as palmitic and stearic acids promote IL-1β release from bone marrow-derived macrophages of rodents and humans, respectively (Wen et al., 2011; L’homme et al., 2013), an effect replicated in murine macrophages (Ann et al., 2018). Of note, immune activation by palmitic and stearic acids precisely depends on the NLRP3-inflammasome pathway (Wen et al., 2011; L’homme et al., 2013). Conversely, unsaturated fatty acids, including oleate and linoleate, prevent IL-1β release and are unable to activate the NLRP3-inflammasome pathway in human monocytes/macrophages (L’homme et al., 2013; Sui et al., 2016). Also, a high-fructose diet in mice positively activates the NLRP3-inflammasome pathway and IL-1β release in human macrophage and liver cell lines, which correlates with neutrophil infiltration (Mastrocola et al., 2016; Nigro et al., 2017; Choe and Kim, 2017). Finally, the role of metabolic species regulating the NLRP3-inflammasome and neurodegeneration was recently evidenced by showing that the 25-hydroxycholesterol also activates the NLRP3-inflammasome pathway, promoting progressive neurodegeneration in X-linked adrenoleukodystrophy, a nervous disease with cerebral inflammatory demyelination (Jang et al., 2016). Moreover, the NLRP3 inflammasome pathway has been identified to contribute to PD (Fan et al., 2017; Gordon et al., 2018; Lee et al., 2018), AD and ALS (Heneka et al., 2013; Johann et al., 2015), HD (Glinsky, 2008), as well as to behavioral alterations in mice at later stages, such as anhedonia (Zhu et al., 2017), anxiety (Lei et al., 2017) and depression-like behavior (Pan et al., 2014; Su et al., 2017).

Altogether, the evidence suggests that overnutrition during pregnancy might promote microglia activation, which correlates with peripheral pro-inflammatory profiles and brain abnormalities in the offspring that are related with neurodegenerative susceptibility. We next discuss the role of diet-induced central and peripheral immune training on neurodegeneration.

## Does Central and Peripheral Innate Immune Training by NLRP3 Inflammasome Contributes to Neurodegeneration?

**FIGURE 2:**
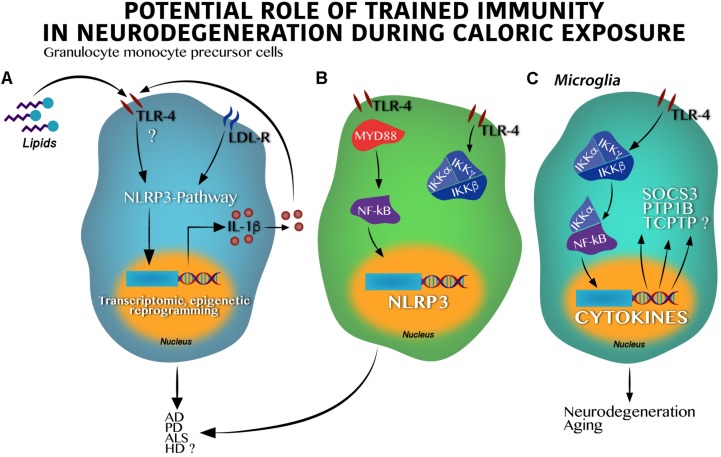
Toll-like receptor 4 activation by hypercaloric diet regulates inflammasome activation in innate cells leading to neurodegeneration. **(A)** In granulocyte monocyte precursor cells lipids activate Toll-like receptor 4 (TLR-4) leading to the inflammasome-pathway (NLRP3) activation, epigenetic, and transcriptomic changes in nucleus and IL-1β synthesis and release. IL-1β itself might stimulate an autocrine loop activating the TLR-4. **(B)** Classic activation of the IKK kinase complex (IKKα and IKKβ) by lipids in granulocyte monocyte precursor cells over supply leads to the NF-κB transcription factor activation and translocation into the nucleus positively regulating the NLRP3 synthesis. Dual activation of the **(A)** and **(B)** pathways result in neuronal death found in AD, PD, HD, and potentially in ALS. **(C)** Activation of the TLR-4 in microglia stimulates the IKK kinase complex following by the NF-κB activation and translocation into the nucleus leading to cytokines, suppressor of cytokine signaling 3 (SOCS3), protein-tyrosine phosphatase 1B (PTP1B) and probably, T-cell protein tyrosine phosphatase (TCPTP) synthesis, which altogether promote neurodegeneration and aging.

Trained immunity is a selective innate immune memory that induces an enhanced response to an inflammatory stimulus in innate immune cells (for instance, microglia), with a much stronger effect than that observed in basal conditions. Trained immunity depends of signal imprinting on transcription factors and epigenetic reprograming (Figure 2A). At this state, microglia in the CNS, or macrophages, leukocytes, natural killer cells, etc., in the peripheral system undergo apparent changes in their morphology, elevated secretion of cytokines and other inflammatory mediators, as well as substantial epigenetic program settings (Netea et al., 2016). Recent reports in different models and also in humans have confirmed robust innate immune training of myeloid cells following a pro-inflammatory challenge (Arts et al., 2018; Bekkering et al., 2018; Christ et al., 2018; Kaufmann et al., 2018; Mitroulis et al., 2018). For instance, exposure to a brief high-fat diet for 3 days promoted the activation of the NLRP3 inflammasome pathway, leading to central immune training, which potentially associates to deficits in long-term memory formation in mice (Sobesky et al., 2016). Also, peripherally applied inflammatory stimuli induce acute immune training in microglia, which correlates with differential epigenetic reprograming that modulates the microglial Tak1 or Hdac1/2 genes. Notably, microglia immune training persists for at least 6 months and increases neuronal death in an animal model of AD and ischemia (Wendeln et al., 2018). These results support the importance of central immune training as a positive regulator of neurodegenerative susceptibility that, during nutritional programing, depends on the NLRP3 inflammasome pathway, as we mentioned before (Christ et al., 2018).

Major advances demonstrating the role of the NLRP3 inflammasome pathway on neurodegenerative susceptibility have been identified in animal models. In fact, it is believed that the NLRP3 inflammasome pathway goes beyond IL-1β synthesis, activating the function of the NF-κB factor and modulating neurodegenerative susceptibility. At first, experimental studies using murine models propose that NF-κB seems to have a role in the development of synaptic plasticity and neuronal survival (Monje et al., 2003), however, over activation of the IKKβ/NF-kB binomial might set the scenario toward a new pro-inflammatory state involving TNF-α through the paracrine actions modulating neuronal survival (Figure 2B). For instance, microglia IKKβ/NF-κB ablation in the hypothalamus reverts cognitive decline in rodents (Zhang et al., 2013), whereas IKKβ/NF-κB activation under long-time exposure to a hypercaloric diet depletes and impairs neuronal differentiation of adult hypothalamic neural stem cells (Li et al., 2012).

In the context of overnutrition, the IKKβ/NF-kB pathway (Figure 2C) seems to activate metabolic-related neurodegenerative markers including the suppressor of cytokine signaling 3 (SOCS3), the tyrosine-protein phosphatase non-receptor type 1 (PTP1B), and potentially, the T-cell protein tyrosine phosphatase (TCPTP) (Milanski et al., 2009). SOCS3 is implicated in neuronal damage during aging (Yadav et al., 2005; DiNapoli et al., 2010), and is also activated during Wallerian degeneration in injured peripheral nerves (Girolami et al., 2010; McNamee et al., 2010). The PTP1B marker leads to neuronal damage in the developing brain (Liu et al., 2019) and is actively implicated in apoptotic neuronal death in a high-glucose *in vitro* model (Arroba et al., 2016). Of note, PTP1B inhibition significantly inactivates GSK-3β-suppressing, amyloid β (Aβ)-induced tau phosphorylation and ameliorates spatial learning and memory in an animal model of AD (Kanno et al., 2016). On the other hand, the TCPTP marker was initially identified as a contributor to leptin resistance in the hypothalamus during obesity in rodents (Loh et al., 2011) and as a modulator of the homeostasis of body glucose (Dodd et al., 2015, 2018). Recently, TCPTP was reported to modulate cytokine release by the expression of connexin 30 (CX30) and connexin 43 (CX43) from astrocytes in a reciprocal interplay with PTP1B following LPS administration (Debarba et al., 2018). In fact, connexin43 or pannexin1 in astrocytes has been demonstrated to promote activation of the inflammasome in glial cells (Yi et al., 2016) and, its blockade with boldine, inhibited hemichannel activity in astrocytes and microglia without affecting gap junctional communication in culture and acute hippocampal slices. Also, when tested in animal models, AD mice with long-term oral administration of boldine prevented the increase in glial hemichannel activity, astrocytic Ca^2+^ signal, ATP and glutamate release which in turn, alleviated hippocampal neuronal suffering (Yi et al., 2017). These evidences allow us to propose that the IKKβ/NF-kB pathway activation during overnutrition integrates the PTP1B – TCPTP cross-talk which might be potentially implicated in neuronal survival. Together, given that overnutrition is associated to immune training by regulating the NF-κB activation linked to the NLRP3 inflammasome pathway (Christ et al., 2018), we propose that maternal nutritional programing by exposure to hypercaloric diets during pregnancy might set microglia immune training by activation of the IKKβ/NF-kB pathway, amplifying its effects on the SOCS3, the PTP1B, and the TCPTP (Figure 2C). Altogether, these proteins might increase susceptibility to neurodegenerative diseases in the offspring at later stages.

**FIGURE 3:**
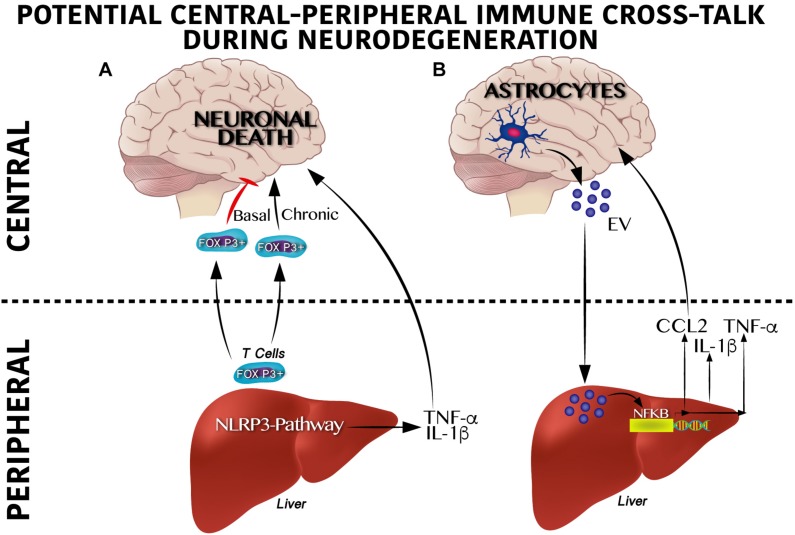
Cross-talk between peripheral and central immune system on neurodegenerative susceptibility. **(A)** Peripheral immune system regulates neurodegeneration. Under a physiological scenario, Foxp3 + T cells are released from the liver to circulation reaching the central nervous system (CNS) positively modulating neuronal survival, whereas soon after a chronic activation Foxp3 + T cells become overactive promoting neuronal death. Likewise, activation of the inflammasome pathway in the liver produces IL-1β and TNF-α which exacerbate neurodegeneration. **(B)** Central immune system regulates neurodegeneration. Astrocytes secrete extracellular vesicles (EV) to the circulation reaching the liver and positively modulating the NF-κB transcriptional activation program, which includes IL-1β, TNF-α and CCL2 synthesis and release. This pro-inflammatory profile has been reported to contribute centrally to neuronal damage.

Potential molecular mechanisms that regulate peripheral immune training linked to NLRP3 inflammasome and neurodegeneration are being elucidated. Qiao et al. (2018) identified that hepatic NLRP3 inflammasome inhibition by *in vivo* siRNA administration decreases the TNF-α, IL-β, IL-12, and IL-18 serum levels. Of note, inhibition of the hepatic NLRP3 inflammasome partially prevents dopaminergic neuronal death in the substantia nigra of a murine Parkinson model by decreasing the pro-inflammatory profile in plasma (Qiao et al., 2018). It seems that immune activation, at least for viral infections, depends on CXCR6, a chemokine receptor of hepatic NK cells involved in the persistence of cellular memory (Netea et al., 2011). This suggests a potential direct link between peripheral immune training in the liver and neuroinflammation that leads to neurodegeneration in the CNS (Figure 3A).

Moreover, initial central immune training and its cross-talk with peripheral immunity also seems to be involved in neurodegeneration. For instance, recently, a substantial accumulation of peripheral FOXP3 + regulatory T cells in the mouse brain following ischemic stroke (Ito et al., 2019) was described. Of note, central FOXP3 + regulatory T cells accumulation inhibits neurotoxic astrogliosis by releasing amphiregulin, a low-affinity ligand of the epidermal growth factor receptor (Ito et al., 2019). Central FOXP3 + regulatory T cell accumulation appears to have a binary role on neuronal survival (Figure 3A). Transient depletion of FOXP3 + regulatory T cells decrease amyloid-β plaque accumulation, reverts the neuroinflammatory response and improves cognitive performance in the 5XFAD AD mouse model, therefore is beneficial under acute central inflammation (Baruch et al., 2015). Conversely, peripheral immune training in the liver might also be regulated by central immune inflammation (Figure 3B). Initial reports show that extracellular vesicles released from astrocytes and microglia during central immune training might be implicated in the peripheral-central immune cross-talk (Wang et al., 2017; Yang et al., 2018). For instance, in murine models, intracerebral injection of IL-1β promotes the release of extracellular vesicles from astrocytes, leading to leukocyte migration into the brain and regulation of the liver cytokines profile by inhibition of the peroxisome proliferator-activated receptor α (Dickens et al., 2017). It is important to mention that extracellular vesicles released by central immune activation depend on glutamine modulation by glutaminase enzyme activation (Dickens et al., 2017; Wang et al., 2017; Wu et al., 2018). In fact, substantial programing of metabolic related pathways coordinates innate immunity. For instance, M1 pro-inflammatory state in macrophages depends largely on glycolytic metabolism, impairment of oxidative phosphorylation and disruption of the Krebs cycle; glycolysis activation during immune training was recently reported to be dependent on the mevalonate pathway (Bekkering et al., 2018). Conversely, macrophages M2 phenotype, which are involved in tissue repair and homeostasis, are dependent on the Krebs cycle (Netea et al., 2011). These results suggest that a shift from oxidative phosphorylation toward glycolysis seems to be a potential metabolic node to regulated immune training. In line with this, the Akt/mTOR/HIF-1α–dependent pathway and the brain neutral sphingomyelin hydrolase 2 have been reported to be essential for trained immunity (Cheng et al., 2014; Saeed et al., 2014; Dickens et al., 2017).

A pro-inflammatory profile has been demonstrated to contribute to four major neurodegenerative pathologies and either suppressing or ameliorating this inflammatory process can have a good outcome for these diseases. According to this, we next discuss four of the major neurodegenerative pathologies in which inflammation has been proven to play a key role.

## Central and Peripheral Inflammatory Nodes in Neurodegenerative Diseases

### Active Immunity in Alzheimer Disease (AD)

Alzheimer disease is the most common type of senile dementia. Worldwide, approximately 50 million people are living with dementia and this number will triple in the next 40 years (Venigalla et al., 2015). AD is characterized by Aβ aggregation and intracellular bundles of hyperphosphorylated Tau protein (Colonna and Butovsky, 2017), which contribute to cognitive decline (Heneka et al., 2015).

High levels of TNF-α have also been identified in murine models of AD that resemble the classical behavioral abnormalities of the disease in humans (Gabbita et al., 2012; Kinney et al., 2018). Blocking the TNF-α pathway decreases central immune infiltration and prevents AD neurochemical and behavioral phenotypes (Tweedie et al., 2012; Prasad Gabbita et al., 2015), suggesting that central–peripheral cross-talk may be implicated in the disease. Notably, experimental studies using transgenic AD models have demonstrated that non-steroidal anti-inflammatory drugs (NSAIDs) can reduce AD pathology (Kinney et al., 2018). In addition, not only vertebrates seem to have dysregulation of the immune system during AD: In a *D. melanogaster* AD-like model, there is an upregulation of all five antimicrobial peptides (AMPs) related to inflammatory genes, suggesting the activation of innate immunity in AD-like flies. To counteract this neuroinflammation, they fed the flies with *Gardenia jasminoides* extracts (iridoid glycosides and aglycones, such as gardenoside, crocin, geniposide, and genipin) whose components can rescue cognitive deficits in a memory impairment mouse model (Nam and Lee, 2013) and show neuroprotective effect in cell culture (Sun et al., 2014). This experiment demonstrated that even though these extracts did not alter the Aβ concentration, they regulated the expression of immune-related genes (Drosomycin, Diptericine, Attacin, Cecropin, Mtk) in the brain of the flies and ameliorated memory deficits (Wei-Wei et al., 2017). The amelioration of memory loss and the counteraction of inflammation occurs through the modulation of the activity of acetylcholine esterase and the regulation the genes in apoptosis for neuroprotection, which have been recently accounted for in glycosides and aglycones present in *Gardenia jasminoides* extracts (Sun et al., 2014). Moreover, the robust effect of central-peripheral immune activation on AD-like neuropathology is also simulated using prenatal immune activation by the bacterial endotoxin lipopolysaccharide (LPS) or polyinosinic:polycytidylic acid (PolyI:C) (Chen et al., 2011; Krstic et al., 2012; Giovanoli et al., 2015, 2016) these findings suggest that prenatal immune activation may be an important environmental risk factor that can cause hippocampus-related cognitive and synaptic deficits in the absence of chronic inflammation across aging.

Initial reports in humans have identified a proinflammatory profile of cytokines such as TNF-α, IL-6, and IL-1β in the prefrontal cortex, hippocampus and serum of AD subjects (Cacabelos et al., 1994; Mrak and Griffin, 2005; Calsolaro and Edison, 2016). In fact, the risk for progression from mild cognitive impairment to AD and cognitive decline is higher in subjects with elevated IL-6 and TNF-α levels in the cerebral spinal fluid (Heneka et al., 2015). In the 1980s, when neuroinflammation was described in AD and immune-related cells where described to be close to Aβ plaques, several large epidemiological and observational studies were published concluding that long-term use of NSAIDs showed protective qualities against developing AD. However, recent studies have identified variable outcomes and no convincing evidence regarding the positive effect of NSAIDs in AD (Kinney et al., 2018). Despite these experimental evidences, it is still unknown whether elevated levels of IL-1β, IL-6 and TNF-α lead directly to neurodegeneration in humans (Figure 1).

### Active Immunity in Parkinson’s Disease (PD)

Parkinson’s disease is a the second most common neurodegenerative condition, affecting about 1.6% of people over 60 years old; it acts by promoting dopaminergic neuronal death in the substantia nigra pars compacta (Bassani et al., 2015). PD shows intraneuronal aggregates (called Lewy bodies) formed by α-synuclein that contribute to dopaminergic neuronal death in the basal ganglia, leading to the classical parkinsonian motor symptoms (De Virgilio et al., 2016). Neurodegeneration in PD is associated with the activation of microglia (Heneka et al., 2015), increased TNF-α, IL-1β, and IL-6 levels in the midbrain, serum and cerebral spinal fluid (Hofmann et al., 2009; Montgomery and Bowers, 2012) and activation of astrocytes (Wang et al., 2015).

Potential systemic triggers of immune activation in PD have identified that maternal immune activation in murine models reproduces the neurochemical and neuropathological symptoms of the disease (Wang et al., 2009; Vuillermot et al., 2012). Also, TNF-α and IL-6 levels correlate with dopaminergic neuronal death in the PD brain – it seems that both cytokines show a dual role on neurodegeneration and neurophysiology (Figure 1). Another *in vivo* example in which neuroinflammation is involved in neurodegeneration is the *D. melanogaster* Rotenone PD-like model. Lakkappa et al. (2018) used a new compound, PTUPB [(4-(5-phenyl-3-3-3-(4-trifluoromethyl-phenyl)-ureido-propyl-pyrazol-1-yl)-benzenesulfonamide)], that inhibited neuroinflammation and in turn prevented the loss of dopaminergic neurons, slightly ameliorating the PD-like pathology.

Similar to the animal models, post-mortem PD patients show infiltration of T-lymphocytes into the substantia nigra (Montgomery and Bowers, 2012). The immune hypothesis as a tentative cause of PD is still active given that, similar to AD genetic risk factors, PD involves the LRRK2, NR4A2, PARK7, and CD74 genes, which are capable of regulating immune function and microglial activation (Pandey et al., 2009). In summary, the involvement of immune responses in PD is not fully understood, but they influence the inflammation response and seem to affect disease progression.

### Active Immunity in Huntington’s Disease (HD)

Huntington’s disease is an autosomal-dominant neurodegenerative disease caused by a CAG trinucleotide repeat expansion that gives place to a polyglutamine region in the huntingtin protein (HTT). HD patients show a positive pro-inflammatory profile (Figure 1) correlated with the disease’s progression, including increases of IL-1β, IL-6, and TNF-α in the striatum, cerebral spinal fluid and plasma that were also confirmed in mouse models (Rocha et al., 2016). Central immune activation in HD subjects was confirmed using positron emission tomography (PET) and in pre-symptomatic HD-gene carriers (Rocha et al., 2016). Even though pharmacologic immune modulation using the XPro1595 TNF-α inhibitor has shown neuroprotection against the cytokine-induced neurotoxicity in primary R6/2 neurons and human neurons derived from iPSCs of HD patients (Hsiao et al., 2014), the information available is contradictory based on one report describing that neuroinflammation seems a consequence rather than a cause of neurodegeneration in late HD (Vinther-Jensen et al., 2016).

### Active Immunity in Amyotrophic Lateral Sclerosis (ALS)

Amyotrophic lateral sclerosis is the most prevalent kind of motor neuron disease in adults, affecting 4–6 per 100,000 people, and is considered a fatal neurodegenerative disease (Filippi and Agosta, 2016). ALS is characterized by the degeneration of motor spinal neurons, brain stem and primary motor cortex mainly associated to a neuroinflammatory response by central (microglial) and peripheral (T-cells) immune activation and infiltration into the affected brain regions (Evans et al., 2013; Filippi and Agosta, 2016). Central immune activation also occurs in patients with either sporadic or familiar ALS, as well as in transgenic models of the disease (Geloso et al., 2017). Immune activation has also been reported in a model of ALS in *D. melanogaster*, in which the flies were supplemented with potent anti-inflammatory natural extracts of *Withania somnifera* (Wse) and *Mucuna pruriens* (Mpe), which significantly rescued climbing impairment (De Rose et al., 2017). Also, ALS patients and mouse models of the disease have elevated levels of TNFα, IL-6, and IL-1β in blood and cerebrospinal fluid. In addition to TNF-α upregulation in ALS, TNFR1, and TNFR2 transcripts have also been found to be overexpressed in ALS patients compared with non-neurological controls (Han et al., 2015; Tortarolo et al., 2017). Similar to Alzheimer, Parkinson and Huntington, modulation of inflammatory cytokine–dependent pathways prevent neuronal death in ALS models. As we commented previously, the contribution of TNFα or IL-6 (Figure 1) as etiological causes of ALS pathophysiology remains controversial because of their physiological functions or dual role on the central and peripheral immune activation or as a neurotrophic factors (Tortarolo et al., 2017).

Altogether, these results propose a very new and not-yet-understood pathway that integrates metabolism, central and peripheral immune training and cross-talk which might actively modulate neurodegeneration.

## Conclusion

Maternal nutritional programing and immune training in microglia or peripheral innate cells are complex and require a profound integration of glycolytic and oxidative metabolism as well as the reprograming of molecular signatures that modulate susceptibility to neuronal damage at earlier stages of development in the offspring. Peripheral or central innate immune training during maternal nutritional programing might integrate a significant node that regulates neurodegenerative susceptibility in the offspring. Potential integration of the NLRP3 inflammasome and the IKKβ/NF-kB pathways in microglia at early stages, and peripheral or central immune cross-talk at later stages, including the FOXP3 + regulatory T cells or the extracellular vesicles, might actively regulate neuroinflammation and neuronal death/survival. This information allows us to propose that maternal nutritional programing leads to peripheral or central immune training, favoring neurodegenerative susceptibility.

## Author Contributions

MC-T, LM-M, RM-R, AC-M, and DR-P: conceptualization and writing – review and editing. AC-M and DR-P: supervision and visualization.

## Conflict of Interest

The authors declare that the research was conducted in the absence of any commercial or financial relationships that could be construed as a potential conflict of interest.
